# Predictors and patterns of gambling behaviour across the COVID-19 lockdown: Findings from a UK cohort study

**DOI:** 10.1016/j.jad.2021.10.117

**Published:** 2022-02-01

**Authors:** Meg Fluharty, Elise Paul, Daisy Fancourt

**Affiliations:** a Department of Behavioural Science and Health, University College London, United States

**Keywords:** COVID-19, Lockdown, Gambling, Coping, Risk behaviours

## Abstract

The negative impact of the COVID-19 pandemic on the economy has raised concerns about negative coping behaviours to offset financial losses. We used a subset of a longitudinal study of UK adults (*N* = 19,963) to examine a range of predictors of (i) gambling during the first strict lockdown, (ii) gambling more frequently during this strict lockdown compared to before lockdown, and (iii) continued increased frequency of gambling during the relaxation of restrictions. Results from logistic regressions indicated that amongst other factors, those with progressively lower levels of education, were stressed due to boredom, frequently drank alcohol, and had high risk-taking tendencies were more likely to gamble during strict lockdown. Individuals who were more likely to have increased their frequency of gambling during strict lockdown compared to before the lockdown were stressed by boredom, employed, frequently drank alcohol, and had depression and anxiety, whilst men and current smokers were less likely. As lockdown restrictions eased, individuals of ethnic minority backgrounds, who were current smokers, and with lower educational attainment were more likely to continue gambling more than usual. Findings contribute to knowledge of who is most at risk for increasing their gambling during the COVID-19 pandemic.

## Highlights

• Examined predictors and changes in gambling behaviour over the course of the lockdown in the UK.
• 9.2% of the study population increased their gambling during strict lockdown in the UK.
• Ethnic minority status, smoking, and lower education levels were risks for continued increased gambling.

## 1 Introduction

In early 2020 a national lockdown order was issued in the United Kingdom (UK) to reduce the spread of COVID-19 and protect healthcare services. Those who had tested positive or were showing symptoms were required to completely self-isolate from others, while those potentially exposed to the virus were ordered to quarantine. One major implication of the pandemic has been major economic upheaval, with the resulting economic recession expected to be more severe than the 2008 recession (World Trade Organisation, 2020). Recessions have previously been shown to be bad not just for individual finances, but also in terms of increases in maladaptive coping behaviours such as gambling (Horváth and Paap, 2012; Olason et al., 2017). Gambling can broadly be defined as placing something of value at risk in hopes of gaining something of greater value, with typical forms including wagering at casinos, lotteries, betting on sporting events, and card games (Potenza et al., 2002). Gambling is common in the UK; before the lockdown, in December 2019, nearly half of the UK adult population had participated in some form of gambling in the past four weeks (Gambling Commission, 2020). Evidence from previous financial crises have shown increases in gambling participation, and those using gambling as a coping strategy were eight times more likely to exhibit problem gambling compared to more stable economic circumstances (Economou et al., 2019; Olason et al., 2015).

In the UK, there has been concern that the COVID-19 pandemic could lead to increases in gambling behaviour as a way for individuals to deal with the unprecedented social isolation and financial stress (Håkansson, 2020). During the UK's lockdown, betting shops and casinos were ordered to close, but lottery tickets could still be purchased in essential shops (i.e. supermarkets) (Gambling Commission, 2020). Whilst some forms of gambling became more inaccessible during lockdown, online gambling remained widely available (Håkansson, 2020). A US-based internet provider (Verizon) reported a 75% increase in online activity during the pandemic (King et al., 2020), with similar rates of increase reported in Italy (King et al., 2020; Perez, 2020). The UK Gambling Commission reported changes in the types of gambling early in the pandemic, with the proportion engaging in online gambling increasing from 26% to 42% in April 2020, and with current gamblers trying one or more new gambling activities for the first time (Gambling Commission, 2020), and Further, moreincreased time and money spent on online gambling sites (Gambling Commission, 2020). Online gambling is particularly concerning as its anonymity and privacy may facilitate the initiation of gambling in individuals who would not normally gamble in-person venues, as well as allowing for greater flexibility in making small bets and experimenting with multiple forms of gambling (Gainsbury, 2013]. Online gambling has therefore been suggested to be the most problematic form of gambling, with higher rates than offline forms and a greater likelihood of pathological use (Ronzitti et al., 2016).

While there are a number of known predictors of gambling behaviour such a male gender (Nelson et al., 2006), risk taking tendencies (Gupta et al., 2006) and substance use (Kranzler & Tinsley, 2004; Mcgrath and Barrett, 2009), it is unknown how these factors may relate to gambling during the COVID-19 pandemic as people were restricted to their homes and may have been facing additional health and financial stressors. Further, much of the literature has focused on problem gambling, so there is little evidence of the predictors of lower-risk gambling within the general population, as the majority of research focuses on harmful levels of gambling (Currie et al., 2006). Yet lower-risk gambling is also a concerning behaviour. Individuals can become subject to financial trouble as they attempt to ‘even’ out their losses, subsequently acquiring debt and needing to further financial resources (i.e. second job) to fund gambling (Langham et al., 2016). Secrecy of gambling habits can lead to breakdowns of family structures and marriage (Langham et al., 2016; Murray, 1993). Additionally, those engaging in risky gambling behaviours may face diminished self-image resulting in social isolation and mental ill health including depression and suicidal thoughts and behaviours (McCormick et al., 1984; Bergh and Kühlhorn, 1994). Substance use disorders are often comorbid with problem gambling as the two activities are often engaged in concurrently (Bergh and Kühlhorn, 1994). While gambling in the UK is regulated, the National Audit Office has reported the Gambling Commission is unlikely to be fully effective in identifying risks and harms to consumers, including identifying when and which consumers may be most vulnerable to harmful gambling (iGaming Business, 2020).

Therefore, it is important to understand factors associated with gambling during the COVID-19 lockdown, and how gambling behaviours may have changed over the course of the pandemic. This will enable the design and delivery of targeted support for individuals who may be at risk for problem gambling. By examining changes in gambling behaviour during the COVID-19 pandemic, we can identify predictors of risk behaviours when individuals are faced with economic hardships, isolation, and heightened levels of boredom. We used a large panel study of adults in the UK to examine three questions related to gambling behaviour during the COVID-19 pandemic. We aimed to (i) identify patterns of sociodemographic and stress predictors of gambling behaviour during the first strict lockdown, (ii) identify factors associated with gambling more than usual during strict lockdown compared to before lockdown, and (iii) identify factors associated with continuing to gamble more frequently as lockdown restrictions were eased. We hypothesised that sociodemographic and stress risk factors associated with increased gambling during lockdown would be similar to those who continued to gamble more frequently following the end of lockdown.

## 2 Methods

### 2.1 Participants

Data were drawn from the UCL COVID-19 Social Study; a large panel survey of the psychological and social experiences of over 75,000 adults (aged 18+) in the UK during the COVID-19 pandemic. The study commenced on 21 March 2020 and involves online weekly data collection until 21 August 2020 and then monthly collection for the duration of the COVID-19 pandemic. Study sampling was not random and therefore is not representative of the UK population. The sample is however well-stratified and was recruited using three primary approaches. First, convenience sampling was used, including promoting the study through existing networks and mailing lists (including large databases of adults who had previously consented to be involved in health research across the UK), print and digital media coverage, and social media. Second, more targeted recruitment was undertaken focusing on (i) individuals from a low-income background, (ii) individuals with no or few educational qualifications, and (iii) individuals who were unemployed. Third, the study was promoted via partnerships with third sector organisations serving vulnerable groups, including adults with pre-existing mental health conditions, older adults, carers, and people experiencing domestic violence or abuse. For full details on the recruitment strategies of the study, visit www.COVIDsocialstudy.org. The study was approved by the UCL Research Ethics Committee [12,467/005] and all participants gave informed consent.

Inclusion criteria for the first two research questions were (i) non-missing data on the first gambling behaviour module which was administered at study week 10 (28 May to 4 June 2020), (ii) non-missing data on risk-taking tendencies at study week 18 (23 July to 30 July 2020), (iii) usable data on demographic variables used for making statistical weights, and (iv) non-missing data on all predictor variables collected concurrently with the first gambling behaviour module. A total of 32,561 started the gambling module, and out of these, 32,559 participants had non-missing data on all 7 of the gambling questions. Of these, 22,894 also had non-missing risk-taking data. Subsequently, those who responded “other/prefer not to say” to gender (*N* = 76), “prefer not to say” to income (*N* = 2377), and “other/prefer not to say” to ethnicity (*N* = 70) were set to missing. This resulted in the exclusion of 2484 participants, as some gave these responses to more than one of these demographic questions, leaving 20,410. An additional 390 were excluded as they did not have a baseline wave used to derive survey weights (although took part in the demographic part of the survey at later waves). A further 57 were excluded because they were missing data on predictor variables assessed at the same time as our gambling module (pattern of missing data can be found in Supplementary Table S1), leaving 19,963 for our first research question. For our second research question examining change in gambling frequency during strict lockdown (28 May to 4 June 2020) compared to before lockdown, we excluded non-gamblers (*N* = 12,937), leaving a total of 7026.

The sample for our follow-up analyses (research question three) was derived from 19,963 participants in the first sample (28 May to 4 June 2020) at study week 10 who had also taken part in study week 20 (30 July to 7 August 2020), when the item on current gambling frequency compared to strict lockdown was administered (*N* = 17,457). Of these 17,457, we further restricted the sample to those reporting increased gambling in week 10 of the study compared to before lockdown, for a total of 556 participants.

### 2.2 Measures

#### 2.2.1 Gambling behaviour

Gambling behaviour during week 11 of the study (28 May to 4 June 2020) was assessed by asking ‘Since lockdown started, have you engaged in any of the following?’ (1) chance-based gambling, (2) skills-based gambling, (3) fixed odds gambling (4) scratch cards, (5) playing a lottery, and (6) other types of gambling/betting. Responses were “not at all”, “a few times”, “1–2x a week”, “most days each week”, and “everyday”. Responses were collapsed to create our first outcome, binary 'any gambling' vs 'none' variable.

Next, change in the frequency of gambling during strict lockdown compared to before lockdown (prior to March 2020) was assessed by asking ‘How does this betting/gambling compare to your usual levels not in lockdown?’. Response options were “less than usual”, “about the same as usual”, “more than usual”, and “I don't do these things”. Responses were collapsed into “decreased or stayed the same” vs “increased” and those indicating they do not gamble were excluded.

Finally, relative frequency of gambling behaviour was assessed again at week 20 (30 July to 7 August 2020): “In the last two months, across June and July, how does your frequency of betting/gambling compare to during strict lockdown in April/May”. Response options were “less than during April/May”, “about the same as during April/May”, “more than during April/May”, and “I haven't done any betting/gambling in June/July.” Responses were collapsed into “same amount or increased” vs “no gambling or decreased" [compared to June/July].

### 2.3 Predictors

#### 2.3.1 Sociodemographic predictors

Seven sociodemographic predictors were included: (1) gender (male vs female), (2) age group (18–29 vs 30–59 vs 60+), (3) ethnicity (white vs ethnic minority groups [including those identifying as Asian, Black, Chinese, Middle Eastern, mixed race or other ethnic group]), (4) employment status (employed vs student vs inactive [disabled, homemaker, retired]) vs unemployed), (5) educational attainment (up to General Certificate of Secondary Education [(GCSE]) [qualifications at age 16] vs A-Levels [or equivalent] or vocational training [qualifications at age 18] vs undergraduate degree vs postgraduate degree), (6) household income (<£16,000 vs £16–29,999 vs £30–59,999 vs >£60,000 per annum), and (7) housing (living alone vs [with others] not overcrowded vs overcrowded [room per person <1]).

#### 2.3.2 Stress predictors

Nine stress predictors were also included: (1) Stress from boredom was measured by participant indication of whether or not this was a source of major stress (none vs present). (2) Alcohol use was collected in number of drinks per week and categorised into none vs low frequency (0–7) vs high frequency (8+). (3) Current smoking status was measured with a single item ‘Do you smoke?’ with responses of ‘non-smoker,’ ‘former smoker,’ ‘current light smoker (9 or fewer a day),’ or ‘current heavy smoker (; King et al., 2020; Perez, 2020; ; Gainsbury, 2020; Ronzitti et al., 2016; Nelson et al., 2006; Gupta et al., 2006; Mcgrath and Barrett, 2009; )’ collapsed into (non-smoker vs former-smoker vs current smoker). (4) Anxiety symptoms during the past week was measured using the Generalised Anxiety Disorder assessment (GAD-7); a well-validated tool used to screen and identify scores indicative of GAD in clinical practice and research (Spitzer et al., 2006). There are seven items with 4-point responses ranging from “not at all” to “nearly every day”. Those scoring ≥10 were categorised as having met GAD criteria. (5) Depression during the past week was measured using the Patient Health Questionnaire (PHQ-9), a standardised instrument for identify scores indicative of depression in primary care (Löwe et al., 2004). The PHQ-9 consists of nine items with 3-point responses ranging from “not at all” to “nearly every day” (Löwe et al., 2004). Those scoring ≥10 were categorised as having major depression. (6) Financial adversity was operationalised by the experience of at least one of three of specific adversities (whether participants had lost their job or been unable to work; had been unable to pay their bills/rent/mortgage; or had experienced a major cut in household income). (7) Financial worries were categorised as present if they had indicated that loss of job/employment or finances were a major stressor. (8) Isolation status due to the COVID-19 pandemic was measured by number of days participants had left the house in the past week (fully isolating [<1], not isolating [1+]). (9) Risk-taking tendencies were measured with one item from the Dohmen Risk Taking Scale (Dohmen et al., 2011). Respondents rated the extent to which they generally see themselves as a person who is fully prepared to take risks on an 11-point scale from “not at all willing to take risks” to “very willing to take risks”. Behavioural validity has been previously established using a laboratory-based task involving a choice between a safe or a riskier lottery option to win money (Dohmen et al., 2011). In the current study, scores of 0–5 were coded as low risk taking and 6+ as high risk taking.

### 2.4 Analysis

First, we used logistic regression to identify sociodemographic and stress predictors of the presence of any gambling behaviour during strict lockdown whilst mutually adjusting for each predictor. Second, we used logistic regression to examine predictors of gambling more compared to less frequent or no change during strict lockdown (28 May to 4 June 2020) compared to before lockdown began (March 2020), with less frequent or no change in gambling as the reference group. Finally, we used logistic regression to examine factors associated with continuing to gamble more over the past two months than usual as lockdown restrictions eased in individuals who had increased gambling during the initial months of lockdown relative to pre-lockdown. To account for the non-random nature of the sample and to increase representativeness, data were weighted to the proportions of age group, gender, country within the UK, and educational level on the basis of Office for National Statistics (ONS) data (Office for National Statistics, 2020). These cross-sectional weights were created at baseline using the Stata user-written command ‘ebalance’ (Hainmueller and Xu, 2013).

## 3 Results

### 3.1 Sample characteristics

Sample characteristics (both unweighted and weighted) for the first research question are shown in Table 1. Half (48.9%) of participants in the weighted sample were female, 6.7% were from ethnic minority groups, 43.1% aged 30–59, and 22.1% were educated to the undergraduate degree level. Characteristics of gamblers during strict lockdown (second research question) and for the follow-up sample are presented in Supplementary Tables S2 and S3.

**Table 1:** Sample demographic characteristics weighted and unweighted figures (*N* = 19,963).

		**Unweighted (%)**	**Weighted (%)**
Gender	Female	73.7	48.9
Age group	18–29	4.1	6.5
	30–59	52.0	43.1
	60+	42.0	51.4
Ethnicity	Ethnic minority groups	3.4	6.7
Employment	Employed	56.1	50.0
	Student	2.0	3.0
	Inactive	40.2	46.0
	Unemployed	1.8	2.1
Education	Postgraduate degree	27.8	14.5
	Undergraduate degree	42.2	22.1
	A levels or vocational training	16.5	31.2
	Up to GCSE	13.5	32.2
Household income	< £16,000	15.2	20.2
	£16,000- £29,999	26.5	30.6
	£30,000-£59,999	35.1	32.2
	> £60,000	23.2	17.1
Housing	Living alone	23.5	233
	Not overcrowded	70.3	68.6
	Overcrowded	6.2	8.0

During strict lockdown, 14.7% of the total sample had gambled a few times, 12.9% 1–2 times weekly, 1.3% most days, and 0.5% had gambled daily. A detailed description of sample demographics by these gambling frequencies is available in Supplementary Table S4. Of those who had participated in any gambling behaviour at baseline, 79.4% said there was no change in the frequency of gambling during strict lockdown (March to the first week of June 2020) compared to before lockdown, while 11.4% had decreased their gambling, and 9.2.% had increased their gambling frequency. Regarding the types of gambling used, the most common was lottery playing (91.1%), followed by scratch cards (17.5%), chance-based gambling (8.7%), another non-listed form of gambling (4.4%), fixed odds betting (3.6%), and skill-based gambling (e.g., poker or blackjack) (2.2%).

### 3.2 Predictors of any gambling behaviour during strict lockdown (March to early June 2020)

**Fig. 1:**
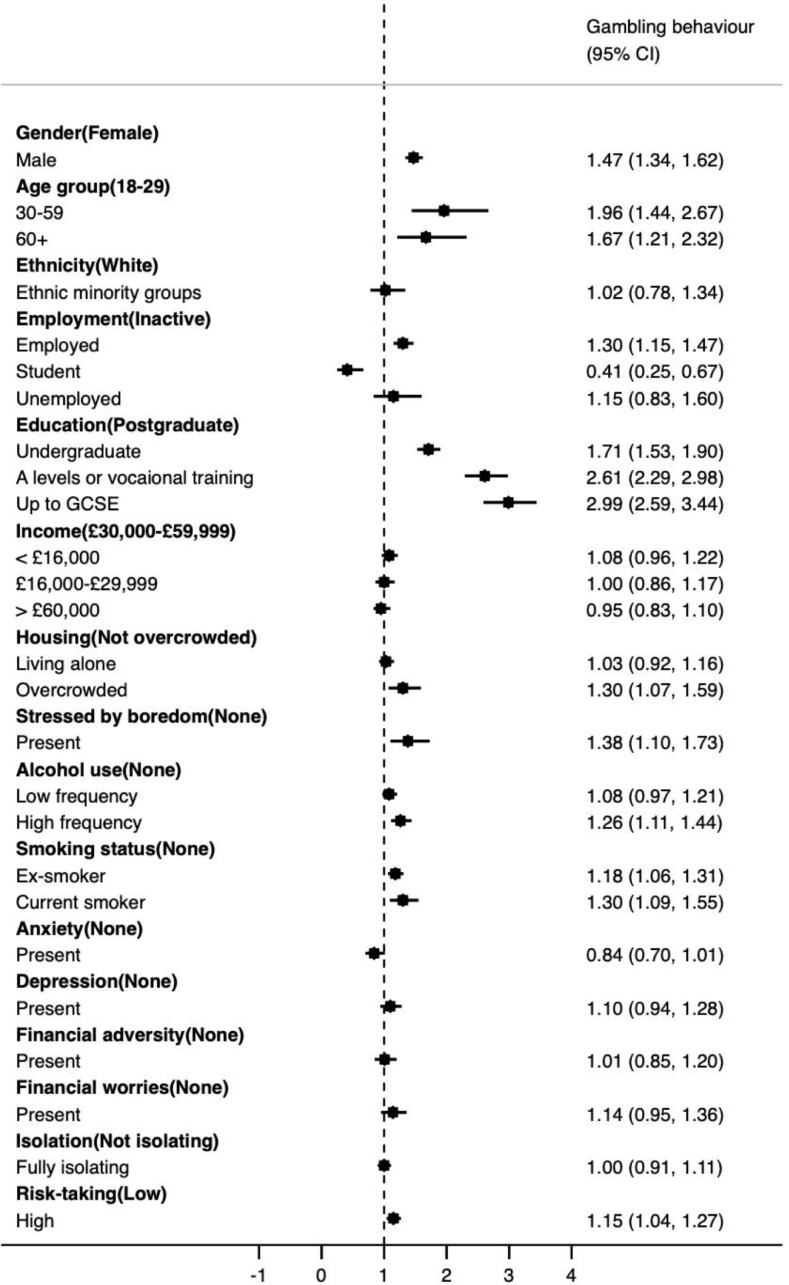
Predictors of any gambling behaviour during strict lockdown (March to early June 2020) (*N* = 19,963) Note. Reference group indicated in parentheses. Outcome is any gambling behaviour versus none during strict lockdown (March to early June 2020).

There were several sociodemographic factors associated with engaging in any gambling behaviour during strict lockdown (Fig. 1). Men (odds ratio [OR]= 1.47; 95% confidence interval [CI]= 1.34, 1.62) were more likely to engage in gambling behaviours than females, as were older adults (ages 30–59; OR= 1.96: 95% CI= 1.44, 2.67; ages 60+: OR= 1.67; 95% CI= 1.21, 2.32) compared to younger adults. Individuals who were employed (OR= 1.30; 95% CI= 1.15, 1.47) were more likely to gamble, while students (OR = 0.41; 95% CI = 0.25, 0.67) were less likely to gamble compared to those inactive in the labour market. Progressively lower levels of educational attainment were associated with higher odds of gambling (up to GCSE: OR= 2.99; 95% CI= 2.59, 3.44; A levels or vocational training: (OR= 2.61; 95% CI= 2.29, 2.98; undergraduate degree: OR= 1.71; 95% CI= 1.53, 1.90) relative to those with a postgraduate degree. Finally, living in overcrowded housing was associated with increased odds of gambling behaviour (OR= 1.30; 95% CI= 1.07, 1.59) compared to those who live with others but in not overcrowded conditions. There were no associations of ethnicity or household income with gambling. Of the stress predictors, stress due to boredom (OR= 1.38; 95% CI= 1.10, 1.73), high frequency alcohol use (compared to non-drinkers, OR= 1.26; 95% CI= 1.11, 1.44), being a former smoker (OR= 1.18; 95% CI= 1.06, 1.31) or current smoker (OR= 1.30; 95% CI= 1.09, 1.55), and high risk-taking tendencies (OR= 1.15; 95% CI= 1.04, 1.27) were all associated with gambling behaviour. There were no associations of depression or anxiety, low frequency alcohol use, financial adversities or worries, or isolation status on gambling.

As a sensitivity analysis, we examined more frequent (1–2x weekly to daily) versus less frequent or none (a few times or none) gambling in place of the binary (any vs none) outcome in these analyses. Results were mostly the same, with some exceptions: being employed, living in overcrowded housing, being a current smoker, and having higher risk-taking tendencies were not associated with increased likelihood of 1–2 weekly to daily compared to less frequent or no gambling (Supplementary Table S5). In contrast, adults aged 30–59 were less likely to gamble 1–2 weekly or daily compared to less frequent or no gambling.

### 3.3 Predictors of gambling more often during strict lockdown (March to early June 2020) relative to before lockdown

Several factors were associated with changes in gambling frequency during compared to before the strict lockdown. Men (OR= 0.77; 95% CI= 0.59, 0.99) and current smokers (OR= 0.64; 95% CI= 0.42, 0.98) were less likely to have increased their gambling frequency (Table 2). Individuals with 10 or more anxiety (OR=1.49; 95% CI= 1.02, 2.19) and depression (OR= 2.11; 95% CI= 1.49, 3.00) symptoms were more likely to say they had been gambling more often during strict lockdown (March to early June 2020) relative to before the lockdown.

**Table 2:** Predictors of an increase in gambling frequency during strict lockdown (March to early June 2020) compared to before lockdown amongst gamblers (*N* = 7026).

		*OR*	*95% CI*		*P*
Gender	Female	–			
	Male	**0.77**	**0.59**	**0.99**	**0.041**
Age group	18–29	–			
	30–59	0.99	0.51	1.91	0.975
	60+	0.73	0.53	1.02	0.062
Ethnicity	White	–			
	Ethnic minority groups	0.72	0.41	1.25	0.239
Employment	Inactive	–			
	Employed	**1.76**	**1.26**	**2.45**	**0.001**
	Student	2.00	0.82	4.84	0.125
	Unemployed	2.15	0.99	4.67	0.053
Education	Postgraduate	–			
	Undergraduate	1.10	0.77	1.56	0.608
	A levels or vocational training	0.86	0.62	1.18	0.346
	Up to GCSE	1.04	0.79	1.38	0.782
Household income	£30,000- £59,999	–			
	< £16,000	1.24	0.85	1.81	0.262
	£16,000- £29,999	0.96	0.70	1.31	0.786
	> £60,000	1.09	0.78	1.52	0.632
Housing	Not overcrowded	–			
	Living alone	0.87	0.63	1.19	0.376
	Overcrowded	1.22	0.81	1.85	0.347
Stress from boredom	None	–			
	Present	**1.72**	**1.09**	**2.71**	**0.020**
Alcohol use	None	–			
	Low frequency	0.97	0.73	1.29	0.849
	High frequency	**1.44**	**1.04**	**1.99**	**0.027**
Smoking status	Non-smoker	–			
	Former smoker	0.90	0.68	1.18	0.434
	Current smoker	**0.64**	**0.42**	**0.98**	**0.039**
Anxiety	None	–			
	Present	**1.49**	**1.02**	**2.19**	**0.040**
Depression	None	–			
	Present	**2.11**	**1.49**	**3.00**	**<0.001**
Financial adversity	None	–			
	Present	1.39	0.96	2.01	0.085
Financial worries	None	–			
	Present	1.15	0.81	1.64	0.437
Isolation	Not isolating	–			
	Fully isolating	0.98	0.76	1.26	0.874
Risk-taking	Low	–			
	High	1.01	0.78	1.32	0.928

Note. Dash indicates reference group. Outcome is gambling increase vs decrease or no change in March to early June 2020 compared to before lockdown.

### 3.4 Predictors of further increases in or sustained gambling frequency during eased lockdown (30 July to 7 August 2020)

amongst the 556 individuals who had increased their gambling during strict lockdown, 267 had further increased or maintained their gambling frequency as lockdown eased. Those from ethnic minority groups (OR= 3.64; 95% CI= 1.23, 10.79), who had educational attainment up to GCSE (OR= 3.14; 95% CI= 1.54, 6.39) or A-levels or vocational training (OR= 2.40; 95% CI= 1.16, 4.97) compared to those with a postgraduate degree, and current compared to non-smokers (OR= 2.72; 95% CI= 1.16, 6.37) were more likely to continue gambling at the same frequency or have increased their gambling frequency as lockdown restrictions eased (Table 3). Students (OR= 0.02; 95% CI = 0.00, 0.28) were less likely to continue gambling at a higher or the same frequency.

**Table 3:** Predictors of a further increase or a sustained increase in gambling frequency during eased lockdown (30 July to 7 August 2020) in those who had increased gambling frequency during strict lockdown (March to early June 2020) (*N* = 556).

		**OR**	**95% CI**		**P**
Gender	Female	–			
	Male	1.48	0.89	2.46	0.128
Age group	18–29	–			
	30–59	2.19	0.62	7.75	0.223
	60+	0.84	0.47	1.50	0.560
Ethnicity	White	–			
	Ethnic minority groups	**3.64**	**1.23**	**10.79**	**0.020**
Employment	Inactive	–			
	Employed	0.97	0.50	1.88	0.926
	Student	**0.02**	**0.00**	**0.28**	**0.004**
	Unemployed	0.72	0.20	2.63	0.621
Education	Postgraduate	–			
	Undergraduate	1.29	0.70	2.38	0.417
	A levels or vocational training	**2.40**	**1.16**	**4.97**	**0.019**
	Up to GCSE	**3.14**	**1.54**	**6.39**	**0.002**
Household income	£30,000- £59,999	–			
	< £16,000	0.88	0.41	1.90	0.746
	£16,000- £29,999	0.66	0.34	1.29	0.222
	> £60,000	1.39	0.72	2.69	0.324
Housing	Not overcrowded	–			
	Living alone	0.97	0.51	1.83	0.920
	Overcrowded	0.70	0.32	1.55	0.379
Stress from boredom	None	–			
	Present	0.64	0.26	1.55	0.320
Alcohol use	None	–			
	Low frequency	0.78	0.43	1.44	0.431
	High frequency	1.38	0.74	2.55	0.310
Smoking status	Non-smoker	–			
	Former smoker	1.23	0.70	2.15	0.464
	Current smoker	**2.72**	**1.16**	**6.37**	**0.021**
Anxiety	None	–			
	Present	0.60	0.28	1.32	0.208
Depression	None	–			
	Present	0.71	0.36	1.38	0.305
Financial adversity	None	–			
	Present	1.14	0.59	2.22	0.698
Financial worries	None	–			
	Present	1.21	0.61	2.41	0.591
Isolation	Not isolating	–			
	Fully isolating	0.67	0.40	1.12	0.123
Risk-taking	Low	–			
	High	0.97	0.58	1.61	0.897

Note. Dashes indicate reference group. Outcome is “same amount or increased” vs “no gambling or decreased" [compared to June/July] as gambling during eased lockdown (30 July to 7 August 2020) compared to strict lockdown.

## 4 Discussion

Using longitudinal data from a large sample of UK adults, we found that several factors related to gambling behaviour and to changes in the frequency of gambling behaviour over time as the pandemic progressed. Men, adults ages 30 years and older, the employed (vs inactive), those with progressively lower levels of educational attainment below a postgraduate degree, people who lived in overcrowded housing, those who cited boredom as a source of stress, drank alcohol frequently, were current or former smokers, and had high risk-taking tendencies were more likely to have gambled during strict lockdown, whilst students were less likely to have done so. Several of these groups were also more likely to have gambled more often during strict lockdown compared to before the lockdown; the employed (vs inactive), those who reported that boredom was a source of stress, and who drank alcohol frequently. In addition, individuals who reported 10 or more anxiety or depression symptoms were also more likely to have increased the frequency of their gambling during strict lockdown. In contrast, current smokers and men were less likely to have increased their gambling frequency during this time than non-smokers and women, respectively. Later in the pandemic as lockdown restrictions eased, several groups who had reported already increasing their gambling frequency during strict lockdown had further increased their frequency of gambling or sustained these increased levels; people from ethnic minority groups, those with lower educational attainment (up to GCSE and those with A-levels or vocational training, compared to a postgraduate degree), and current smokers, whilst students were less likely to have done so.

The predictors of gambling behaviour in our study were consistent with those in previous research. Males typically are more likely to gamble and tend to do so in more harmful ways compared to females (Dowling et al., 2017). Previous work suggests that characteristics being male is thought to be a proxy measure for more risk taking and impulsive behaviours (Dowling et al., 2017). However, our study included a measure of risk-taking tendencies which was associated with increased likelihood of engaging in any gambling behaviour, and still found an association with male gender. Older ages have also been observed to gamble more frequently than younger ages, possibly because it may provide a form of risk taking following a lifetime of financial responsibility in older individuals (McNeilly and Burke, 2001). Indicators of lower socioeconomic position (i.e. educational attainment, income, overcrowding) are more likely to be at risk for gambling, as density of gambling machines and shops tend to be higher in areas of lower socioeconomic position (Raisamo et al., 2019; Barratt et al., 2014). Some individuals may view gambling as a way to level up on their socioeconomic position (Castrén et al., 2018).

Substance use (i.e. high alcohol consumption, smoking) have been linked to gambling generally and at harmful levels (Mcgrath and Barrett, 2009). Consumption of substances such as alcohol and tobacco may reinforce cravings and reward of other addictive behaviours (Mcgrath and Barrett, 2009), as well as a way of coping with gambling losses (Yi, 2012). Smoking has also been associated with habitual forms of gambling (i.e. online gaming over less frequent forms such as sports betting) due to the habitual nature of smoking with the two activities becoming conditioned together (Griffiths et al., 2010). Interestingly, while we found smoking to be associated with gambling overall, smoking was associated with a reduced risk of increasing gambling frequency during lockdown. This may be due to the differences in cue-related behaviour for in-person gambling versus internet gambling. For example, individuals who had conditioned these two behaviours together while visiting casinos or betting sites might not have the same cue-reactivity to smoke when engaging online, with similar declines in gambling were observed when smoking bans were introduced to indoor venues (McGrath and Barrett, 2009).

In our study, a 9.2% of adults reported having increased their gambling frequency during strict lockdown, and 14.1% had continued or further increased their gambling frequency following the easing of lockdown restrictions. Even though restrictions had eased, the period following strict lockdown was marked by concerns about an upcoming economic recession which may have led to increased usage of maladaptive coping strategies to cope with economic insecurities and to alleviate boredom. We found that a number of factors to be associated with having increased gambling frequency during strict lockdown. Previous studies have found weak and inconsistent associations of unemployment with gambling (Arge and Kristjánsson, 2015), and our findings indicated that being employed was associated with increased gambling frequency (compared to those inactive in the labour market). This could have been due to individuals having more time on their hands due to working from home or from increased economic security, however the former was not associated with any of our three gambling outcomes. We did not assess whether those who were employed were on furlough and therefore not actively working during the lockdown, which could have explained the increases in this group. Both boredom as a source of stress and depression symptoms were predictors of gambling in our study and have been associated with problematic gambling to reduce under-arousal in other studies (Mercer and Eastwood, 2010; Blaszczynski et al., 1990). In prior pandemics, systematic review evidence has identified boredom as a source of stress when individuals’ are confined to their homes for unknown periods of time (Brooks et al., 2020). Boredom, in turn is associated with problematic gambling behaviour (Mercer and Eastwood, 2010).

As lockdown eased, those from ethnic minority groups, who had lower levels of educational attainment and were current smokers were most likely to continue or further increase their gambling frequency, indicating these groups may be the most at risk for acquiring problematic gambling habits during lockdown. However, nearly half of those who had increased their gambling ceased gambling altogether at follow-up (30 July to 7 August 2020), suggesting that for them gambling was a transient behaviour during strict lockdown. Similar reports of decreases in online gambling as lockdown eased were reported by the UK Gambling Commission (Gambling Commission, 2020). The Commission also found that lottery and scratch card use increased (as seen in past economic recessions (Horváth and Paap, 2012; Olason et al., 2017)), while other forms of gambling, such as betting, decreased likely due to the pause in English Premier League football and other sporting events (Gambling Commission, 2020). These changes in the types of gambling may be unique to COVID-19, as national stay-at-home orders were paired with job losses, furloughs, and talk of an upcoming economic recession.

This study has a number of strengths including its large sample size, its longitudinal tracking of participants’ gambling behaviour at two points during lockdown, and its rich inclusion of measures on psychological and social experiences during the COVID-19 pandemic. However, there are several limitations. The study is not nationally representative, although it does have good stratification across all major sociodemographic groups and analyses were weighted on the basis of population estimates of core demographics. Whilst the recruitment strategy deliberately over-sampled from groups such as low-income backgrounds, individuals with no or few educational qualifications, and individuals who were unemployed, it is possible that more extreme experiences were not adequately captured. Furthermore, there was a slightly greater risk of dropout amongst individuals engaging in higher levels of gambling and it is therefore possible that the sampling was selective towards infrequent or non-gamblers (which makes our finding of higher levels of gambling and increases in gambling than previous figures more concerning). Additionally, we recognise gender is not binary, and that due to insufficient sample size, had to treat it as such. We therefore realise there is a need for research which acknowledges a wider range of genders. All gambling questions were gathered via self-report on their past gambling each time period as opposed to consistently reporting their current gambling suggesting the results may be subject to recall bias. Additionally, we had good spread across possible responses for each of the measures included in the gambling questions and the sample remained heterogeneous even with attrition. Gambling questions only assessed common types of gambling still available to people during the lockdown and did not measure problematic gambling. We were also unable to examine changes in type of gambling and expenses spent on gambling due to data limitations. Future data collection (i.e., during regional or national lockdowns) may consider examining these factors.

Our findings point to a number of groups at high risk for increasing of gambling during lockdown such as those experiencing symptoms of depression and anxiety, stress due to boredom, who drink frequently, and the employed. In contrast, people from ethnic minority groups, who have lower levels of educational attainment, and who currently smoke may be at risk for further increasing gambling behaviours. These risk groups may be targeted for interventions, by providing information of financial and debt support resources to lower the risk of turning towards gambling. Further, previous work during the COVID-19 pandemic has indicated these groups to be at risk of avoidant coping strategies, which are characterised by attempts to distract oneself and ignore the stressor (e.g. use of substances) (Fluharty and Fancourt, 2020). Therefore, these specific groups may benefit from opportunities to establish more beneficial (and in turn, less harmful) coping strategies, such as social support (Fluharty et al., 2020) or connecting people digitally or through the use of community programmes such as Mutual Aid or schemes such as social prescribing (Moore and March 2020; Saltzman et al., 2020; Tomstad et al., 2017).

## Supplementary material

Supplementary Table S1: Missingness in predictor variables by gambling frequency, unweighted figures (*N* = 19,963)

Supplementary Table S2: Sample characteristics for individuals who reported any gambling during strict lockdown, weighted and unweighted figures (*N* = 7026).

Supplementary Table S3: Sample characteristics for individuals who had increased gambling frequency at baseline and were included in follow-up, weighted and unweighted figures (*N* = 556).

Supplementary Table S4: Sample characteristics by gambling frequency during strict lockdown (March to 4 June 2020) (*N* = 19,963)

Supplementary Table S5. Sensitivity analysis: Predictors of gambling 1–2 weekly or more during strict lockdown (March to early June 2020) (*N* = 19,963)

## Contributors

MF, EP, and DF developed the study concept. MF performed the data analysis and drafted the manuscript. EP and DF provided critical revisions. All authors approved the final version of the manuscript for submission.

## Ethical approval

Ethical approval for the COVID-19 Social Study was granted by the UCL Ethics Committee. All participants provided fully informed consent. The study is GDPR compliant.

## Funding

This COVID-19 Social Study was funded by the 10.13039/501100000279Nuffield Foundation [WEL/FR-000022583], but the views expressed are those of the authors and not necessarily the Foundation. The study was also supported by the MARCH Mental Health Network funded by the Cross-Disciplinary Mental Health Network Plus initiative supported by 10.13039/100007472UK Research and Innovation [ES/S002588/1], and by the Wellcome Trust [221400/Z/20/Z]. DF was funded by the 10.13039/100010269Wellcome Trust [205407/Z/16/Z]. The researchers are grateful for the support of a number of organisations with their recruitment efforts including: the UKRI Mental Health Networks, Find Out Now, UCL BioResource, SEO Works, FieldworkHub, and Optimal Workshop. The study was also supported by HealthWise Wales, the Health and Car Research Wales initiative, which is led by Cardiff University in collaboration with SAIL, Swansea University. The funders had no final role in the study design; in the collection, analysis and interpretation of data; in the writing of the report; or in the decision to submit the paper for publication. All researchers listed as authors are independent from the funders and all final decisions about the research were taken by the investigators and were unrestricted.

## Declaration of Competing Interest

All authors declare no conflicts of interest.

## Appendix Supplementary materials

Image, application 1
