# Supplementary material for: Predictors and patterns of gambling behaviour across the COVID-19 lockdown: Findings from a UK cohort study
Source: J Affect Disord. 2022 Feb 1;298(Pt A):1–8. doi: 10.1016/j.jad.2021.10.117 (PMC8555112; doi:10.1016/j.jad.2021.10.117)
Supplement: Supplementary file 1 [file mmc1.doc]

**Supplementary Material**

***Supplementary Table S1: Missingness in predictor variables by gambling frequency, unweighted figures (N=19,963)***

 **Not at allA few times 1-2x a weekMost days each weekEveryday**%%%%%GenderFemale64.115.517.52.20.7 Male73.114.311.21.00.5Age group18-2984.511.73.20.40.3 30-5968.617.212.41.40.5 60+71.911.814.41.40.6EthnicityWhite73.214.311.20.40.9 Ethnic minority groups70.614.612.91.30.5EmploymentInactive69.116.812.31.30.5 Employed86.09.34.00.50.3 Student72.411.614.11.30.6 Unemployed65.518.113.12.21.1EducationPostgraduate degree80.311.07.90.60.1  Undergraduate degree71.314.512.61.10.5 A levels or vocational training 62.818.316.12.10.7 Up to GCSE 58.717.819.92.51.1Household income< £16,00068.815.812.72.00.7 £16,000- £29,99968.914.614.61.40.6 £30,000- £59,99970.714.513.31.10.6 > £60,00074.214.110.41.10.2HousingNot overcrowded70.814.413.01.20.5 Living alone71.114.412.51.50.5 Overcrowded68.217.112.51.90.3Stress from boredomNone71.014.412.71.30.5 Present62.418.116.42.20.9Alcohol useNone70.714.612.61.50.7 Low frequency71.914.412.31.10.4 High frequency68.514.914.51.60.6Smoking statusNon-smoker73.713.611.31.00.4 Former smoker65.715.616.21.80.7 Current smoker58.820.616.82.51.4AnxietyNone70.714.413.11.30.5 Present70.416.011.21.70.6 Missing85.77.17.10.00.0DepressionNone71.214.213.01.20.5 Present68.716.212.51.90.7 Missing66.716.716.70.00.0Financial adversityNone71.314.312.81.20.5 Present65.517.514.01.91.1Financial worriesNone71.414.112.81.30.5 Present63.919.314.01.61.2IsolationNot isolating70.015.013.21.40.5 Fully isolating72.014.012.41.10.6 Missing93.00.07.00.00.0Risk-takingLow71.214.312.81.30.5 High69.815.213.01.40.6

***Supplementary Table S2: Sample characteristics for individuals who reported any gambling during strict lockdown, weighted and unweighted figures (N=7,026).***

**Unweighted (%)Weighted (%)**GenderFemale67.241.2Age group18-292.43.5 30-5956.746.0 60+40.950.5EthnicityEthnic minority groups3.05.8EmploymentEmployed59.852.4 Student1.00.9 Inactive37.044.4 Unemployed2.22.3EducationPostgraduate degree20.99.6 Undergraduate degree41.619.5 A levels or vocational training20.434.5 Up to GCSE 17.136.4Household income< £16,00015.820.4 £16,000- £29,99927.231.7 £30,000-£59,99935.732.7 > £60,00021.315.2HousingLiving alone32.223.8 Not overcrowded 70.568.2 Overcrowded7.38.0Note. Sample used for research question two; analysis examining changes in gambling frequency during strict lockdown (March to early June 2020).

***Supplementary Table S3: Sample characteristics for individuals who had increased gambling frequency at baseline and were included in follow-up, weighted and unweighted figures (N=556).***

 **Unweighted (%)Weighted (%)**GenderFemale73.650.8Age group18-294.15.8 30-5967.158.4 60+28.835.8EthnicityEthnic minority groups4.06.2EmploymentEmployed70.566.0 Student1.62.1 Inactive25.027.7 Unemployed2.94.3EducationPostgraduate degree22.811.3Undergraduate degree42.122.1A levels or vocational training17.630.4Up to GCSE 17.536.2Household income< £16,00016.223.7 £16,000- £29,99923.423.9 £30,000-£59,99937.434.1 > £60,00023.018.4HousingLiving alone23.020.9 Not overcrowded 65.365.8 Overcrowded11.713.3Note. Follow up sample used for research question three; analysis examining changes in gambling patterns as lockdown eased (30 July to 7 August 2020).

***Supplementary Table S4: Sample characteristics by gambling frequency during strict lockdown (March to 4 June 2020) (N=19,963)***

 **Not at allA few times1-2x a weekMost days each weekEveryday**%%%%%GenderFemale64.015.617.52.20.7 Male73.014.311.31.00.5Age group18-2984.511.73.20.40.3 30-5968.617.212.41.40.5 60+71.811.914.41.40.6EthnicityWhite73.314.111.20.40.9 Ethnic minority groups70.614.613.01.30.5EmploymentInactive72.411.714.11.30.6 Employed69.116.812.31.30.5 Student86.09.34.00.50.3 Unemployed65.418.213.12.21.1EducationPostgraduate degree80.311.18.00.60.1 Undergraduate degree71.314.512.61.10.5 A levels or vocational training62.818.316.12.10.7 Up to GCSE 58.617.820.02.51.2Household income< £16,00068.715.812.72.10.7 £16,000- £29,99968.814.614.61.40.6 £30,000- £59,99970.614.513.31.10.6 > £60,00074.114.110.51.10.2HousingNot overcrowded70.814.513.11.20.5 Living alone71.014.412.61.50.5 Overcrowded68.217.112.51.90.3Stress from boredomNone71.014.512.81.30.5 Present62.418.116.42.20.9Alcohol useNone70.614.612.61.50.7 Low frequency71.814.512.31.10.4 High frequency68.414.914.51.60.6Smoking statusNon-smoker73.613.711.31.00.4 Former smoker65.715.616.21.80.7 Current smoker58.820.716.72.51.4AnxietyNone70.714.413.11.30.5 Present70.316.111.21.70.6DepressionNone71.114.313.01.20.5 Present68.616.312.51.90.7Financial adversityNone71.214.312.81.30.5 Present65.517.614.01.91.1Financial worriesNone71.414.112.81.30.5 Present63.819.414.01.61.2IsolationNot isolating70.015.013.21.40.5 Fully isolating72.014.012.41.10.6Risk-takingLow71.114.312.81.30.5 High69.815.213.01.40.6Note. Sample used for research question one; analysis examining predictors of any gambling behaviour during strict lockdown (March to early June 2020).

***Supplementary Table S5. Sensitivity analysis: Predictors of gambling 1-2 weekly or more during strict lockdown (March to early June 2020) (N=19,963)***

***OR95% CIP***GenderFemale--    Male**1.501.341.67<0.001**Age group18-29--    30-59**0.240.130.45<0.001** 60+**1.060.911.240.427**EthnicityWhite--    Ethnic minority groups1.080.771.520.649EmploymentInactive--    Employed1.040.891.210.650 Student**0.440.200.950.037** Unemployed1.090.731.610.686EducationPostgraduate--    Undergraduate**1.691.461.95<0.001** A levels or vocational training **2.341.982.78<0.001** Up to GCSE **2.902.443.45<0.001**Household income£30,000- £59,999--    < £16,0000.900.741.090.271 £16,000- £29,9991.020.881.180.816 > £60,0000.900.751.070.228HousingNot overcrowded--    Living alone0.980.841.130.751 Overcrowded1.100.861.410.454Stress from boredomNone--    Present**1.371.031.800.028**Alcohol useNone--    Low frequency1.010.881.160.902 High frequency**1.221.031.430.018**Smoking statusNon-smoker--    Former smoker**1.231.071.400.003** Current smoker1.220.981.520.069AnxietyNone--    Present0.820.641.040.101DepressionNone--    Present1.090.891.330.407Financial adversityNone--    Present1.100.891.350.375Financial worriesNone--    Present0.980.781.230.853IsolationNot isolating--    Fully isolating0.990.881.130.932Risk-takingLow--    High1.100.971.240.147Note. Dash indicates reference group. Gambling behaviour outcome variable was coded 1-2 weekly to daily vs a few times or none
